# Placebo: Physician, Heal Thyself

**DOI:** 10.1371/journal.pmed.0020388

**Published:** 2005-11-29

**Authors:** Arunachalam Kumar, C. Jairaj Kumar

**Affiliations:** **1**Kasturba Medical CollegeMangalore, KarnatakaIndia

Kudos to Miller et al. [1] for exposing the seamy side of medical research. No matter how positive the results of drug effectiveness, as demonstrated through against-placebo comparison studies, the fact remains that the ends do not justify the means.

To knowingly and willingly induce a patient to trust in the pharmacotherapeutic effectiveness of a phony drug not only trivializes the patient's intelligence, but also devalues the mentor status of the treating physician.

The role of placebo therapy, though quite dramatic at times, does not sanctify the manner in which large-scale research studies pit one group of falsely guided patients or volunteers against another. The medical and pharmacological world must relegate blind new drug–placebo comparison studies to the back burner. Medical journals, too, have a major role in publishing papers that contain misinformation and that mislead. The psychosomatic pharmacokinetics of drugs can be tested or evaluated through much less dubious means than placebo-based research.

The medical profession has an onus to constantly and continuously present itself as an educated partner of the patient in the treatment of disease and ill health: we are quite positive that not a single volunteer would agree to participate in any placebo–new drug study, if informed of the patently false and fake nature of the research protocols.

There is no denying the fact that placebo therapy has a role in fighting disease, but too much nearly criminal injustice is perpetuated in the form of misinformation in placebo-based research studies. The sooner medical professionals and drug manufacturers proscribe placebo-based research, the better for all.

We congratulate the authors of this Policy Forum [1] for bringing to light this rather dark side of research, and hope that the debate engendered will result in re-establishing the physician as a trusted confidant of the trusting patient.
